# Elucidating transmission dynamics and host-parasite-vector relationships for rodent-borne *Bartonella* spp. in Madagascar

**DOI:** 10.1016/j.epidem.2017.03.004

**Published:** 2017-09

**Authors:** Cara E. Brook, Ying Bai, Emily O. Yu, Hafaliana C. Ranaivoson, Haewon Shin, Andrew P. Dobson, C. Jessica E. Metcalf, Michael Y. Kosoy, Katharina Dittmar

**Affiliations:** a Department of Ecology and Evolutionary Biology, Princeton University, Princeton, NJ, United States; b Division of Vector-Borne Diseases, Centers for Disease Control and Prevention, Fort Collins, CO, United States; c Virology Unit, Institut Pasteur de Madagascar, Antananarivo, Madagascar; d Department of Animal Biology, University of Antananarivo, Antananarivo, Madagascar; e Department of Biological Sciences, Graduate Program in Ecology, Evolution and Behavior, University at Buffalo, Buffalo, NY, United States

**Keywords:** *Bartonella* spp., *Rattus rattus*, *Synopsyllus fonquerniei*, Madagascar, Force of infection

## Abstract

*Bartonella* spp. are erythrocytic bacteria transmitted via arthropod vectors, which infect a broad range of vertebrate hosts, including humans. We investigated transmission dynamics and host-parasite-vector relationships for potentially zoonotic *Bartonella* spp. in invasive *Rattus rattus* hosts and associated arthropod ectoparasites in Madagascar. We identified five distinct species of *Bartonella* (*B. elizabethae 1, B. elizabethae 2, B. phoceensis 1, B. rattimassiliensis 1,* and *B. tribocorum 1*) infecting *R. rattus* rodents and their ectoparasites. We fit standard epidemiological models to species-specific age-prevalence data for the four *Bartonella* spp. with sufficient data, thus quantifying age-structured force of infection. Known zoonotic agents, *B. elizabethae 1* and *2,* were best described by models exhibiting high forces of infection in early age class individuals and allowing for recovery from infection, while *B. phoceensis 1* and *B. rattimassiliensis 1* were best fit by models of lifelong infection without recovery and substantially lower forces of infection. Nested sequences of *B. elizabethae 1* and *2* were recovered from rodent hosts and their *Synopsyllus fonquerniei* and *Xenopsylla cheopsis* fleas, with a particularly high prevalence in the outdoor-dwelling, highland-endemic *S. fonquerniei.* These findings expand on force of infection analyses to elucidate the ecological niche of the zoonotic *Bartonella elizabethae* complex in Madagascar, hinting at a potential vector role for *S. fonquerniei*. Our analyses underscore the uniqueness of such ecologies for *Bartonella* species, which pose a variable range of potential zoonotic threats.

## Highlights

• At least five distinct species of *Bartonella* infect *R. rattus* rodents and their ectoparasites in Madagascar.
• Infection dynamics for zoonotic *B. elizabethae* are consistent with an SIS or SIR model in rodent hosts.
• Infection dynamics for non-zoonotic *B. phoceensis* & *rattimassiliensis* are consistent with an SI model in rodent hosts.
• Transmission of *B. elizabethae* and *B. phoceensis* & *rattimassiliensis*, respectiviely, are affected by *S. fonquerniei* & *X. cheopsis* fleas and *Polyplax* spp. lice.
• *S. fonquerniei*/*X. cheopsis* fleas may vector *B. elizabethae* and *Polyplax* spp. lice vector *B. phoceensis/rattimassiliensis*.

## 1 Introduction

*Bartonella* spp. are facultative, intracellular Gram-negative bacteria, which infect the endothelial cells and erythrocytes of a broad diversity of vertebrate hosts. *Bartonella* spp. have been documented infecting a wide range of mammals (rodents, bats, carnivores, ungulates, and humans, among others; Chomel et al., 2009, Kosoy, 2010), as well as some birds and reptiles (Mascarelli et al., 2014, Valentine et al., 2007). At least twelve known species or subspecies of *Bartonella* bacteria are now recognized zoonotic agents (Vayssier-Taussat et al., 2009, Veikkolainen et al., 2014), and two others, *B. bacilliformis* and *B. quintana* (respectively, the causative agents for Carrion’s disease and trench fever) can maintain transmission in human hosts (Vayssier-Taussat et al., 2009). Zoonotic *Bartonella* spp. typically cause fever, lymphadenopathy, skin lesions, cardiopathy, endocarditis, or neuroretinitis in humans (Breitschwerdt and Kordick, 2000, Kosoy et al., 2003), as exemplified by *Bartonella henselae,* the causative agent in Cat Scratch Disease (Welch et al., 1992). Reservoir hosts typically do not experience substantial pathology upon infection, instead hosting the pathogen in an as-of-yet-undefined primary niche, from which bacteria are seeded into the blood stream to establish long-term erythrocytic infections (Dehio, 2001). Among *Bartonella* spp., *B. bacilliformis* (previously identified only in humans and sandfly vectors) is an exception in causing widespread and life-threatening hemolytic anemia in its primary host (Ihler, 1996).

Five of the recognized zoonotic *Bartonella* spp. (*B. elizabethae*, *B. vinsonii* subsp. *arupensis*, *B. grahamii*, *B. washoensis,* and *B. tribocorum*) are associated with rodent reservoirs (Buffet et al., 2013, Kandelaki et al., 2016, Kosoy et al., 2010, Vayssier-Taussat et al., 2016), and others are likely to be described in future research (Veikkolainen et al., 2014). In previous studies, *Bartonella* spp. prevalence declined with increasing rodent weight (indicative of increasing age or health), suggestive of immune clearance mechanisms (Kosoy et al., 2004b, Morway et al., 2008). One study demonstrated antibody reactivity to *Bartonella* spp. antigens in rodent serum samples (Kosoy et al., 2004a, Kosoy et al., 2004b) but, because *Bartonella* spp. were pooled in weight trend analyses, species-specific infection dynamics could not be resolved (Kosoy et al., 2004a, Kosoy et al., 2004b). In non-rodent hosts (i.e. cats and humans), antibody-mediated immune responses to persistent infections appear to be common (Brouqui et al., 2005, Kabeya et al., 2006, Pearce et al., 2006).

Because infection dynamics often vary with the age structure of the host population, reporting of apparent prevalence, the proportion of individual hosts testing positive for a disease of interest, can belie a disease’s foothold in a host community (Heisey et al., 2006). In contrast, *age-class specific* reporting of prevalence data (or, minimally, weight-class grouping of rodent-borne *Bartonella* spp.; Kosoy et al., 2004b, Morway et al., 2008) can be used to estimate the age-specific force of infection (FOI), or the per capita rate at which susceptible hosts become infected (Muench, 1959). Age-specific FOI can then help identify age-classes most critical in driving population-level patterns of transmission for a given infection and elucidate the influence of specific cohorts on the magnitude and timing of epidemics (Long et al., 2010). In the case of zoonotic infections (i.e. rodent-borne *Bartonella* spp.), the ‘spillover force of infection,’ or the force of infection from animals to humans, is a product of its constituent factors: infection dynamics within the reservoir animal population, animal-human contact rates, and the probability of viable human infection given such a contact (Keeling and Gilligan, 2000, Lloyd-Smith et al., 2009).

We investigated the first of these three factors—infection dynamics within the reservoir host—for rodent-borne *Bartonella* spp. in central Madagascar. In particular, we describe the diversity, distribution, and age-structured transmission dynamics of *Bartonella* spp. infecting peridomestic *Rattus rattus* and their associated arthropod ectoparasites (fleas, lice, mites, and ticks). Vector ecology has been largely understudied in *Bartonella* systems (Chomel et al., 2009), though it is thought that most *Bartonella* spp. are transmitted between hosts by diverse hematophagous arthropods (Billeter et al., 2008), ranging from sandflies (Battisttini, 1931) to lice (Swift, 1920) to fleas (Bown et al., 2004, Chomel et al., 1996), among others. The geographical extent of a vector-borne pathogen is determined by the combined range limits of the host, vector, and pathogen (Reisen, 2010). We explored vector dependency and range limits for potentially zoonotic rodent-borne *Bartonella* spp. in Madagascar.

Previous work has reported *B. quintana* in human lice (Sangaré et al., 2014) and novel genotypes of *Bartonella* in bats and ectoparasite bat flies (Brook et al., 2015, Wilkinson et al., 2016) in Madagascar. To our knowledge, our study represents the first published record of *Bartonella* spp. infecting rodents and their arthropods in Madagascar. Because invasive rodents live in close proximity, often sympatry, with humans in Madagascar (Rahelinirina et al., 2010), the records we report here are important for understanding zoonotic risk. With this analysis, we specifically aimed to: (1) identify *Bartonella* spp. infecting peridomestic rats in Madagascar, including genotypes previously known to be zoonotic, (2) model the age-prevalence of specific rodent-borne *Bartonella* genotypes to develop our understanding of the influence of age-structured force of infection on their transmission, and (3) construct a *Bartonella* spp. phylogeny from both rodent hosts and associated ectoparasites to elucidate possible vector relationships in our system.

## 2 Materials and methods

### 2.1 Field sampling

**Fig. 1:**
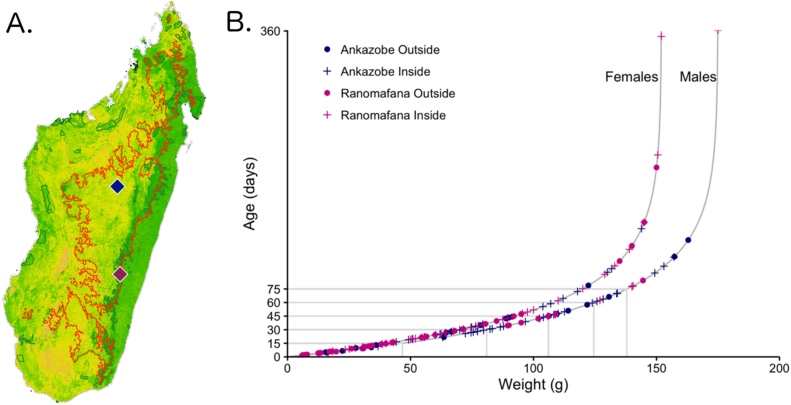
A. Vegetation map of Madagascar (green = forest; yellow = grassland; orange = desert) with 800 m elevation range limit of *S. fonquerniei,* the endemic Malagasy rat flea, highlighted in red. Ankazobe (navy) and Ranomafana (fuschia) sites are marked as diamonds. B. Age-for-weight relationships for captured *R. rattus* as estimated from the von Bertalanffy equation (Ricker, 1979): *W(t)* *=* *W(1 − e^−k(t−t0)^)* in which *W* represents the highest weight class rodent in the data subset (males: 175 g, females: 152 g). Data from Ankazobe sites are depicted in navy (outside sampling sites = circles, inside sampling sites = pluses) and from Ranomafana in fuchsia.

In total, 158 *R. rattus* were trapped and lethally sampled in two different regions in central Madagascar: in peridomestic communities surrounding Ambohitantely Special Reserve, District of Ankazobe, in July 2013 and in both forest and human communities surrounding Ranomafana National Park in June 2014 (Fig. 1A). Kidney tissue samples were collected from all dispatched animals, frozen on site in liquid nitrogen, then transported to −80 °C freezers at the Institut Pasteur of Madagascar, Antananarivo, to await export. Rats in the District of Ankazobe were also flea-combed, and all visible ectoparasites were collected, stored in ethanol, and additionally exported. Of particular note to the analyses presented in this manuscript, the District of Ankazobe is situated firmly on Madagascar’s High Plateau, within the range limits of the endemic *Synopsyllus fonquerniei* rat flea (Rahelinirina et al., 2010), while Ranomafana National Park is located on the peripheral, subtropical boundary of this region. Further details of our field sampling methodology are elucidated in Text S1 (Gerber et al., 2010, Vallan, 2000).

### 2.2 Ethics statement

All field studies were carried out under permit authorization from Madagascar National Parks and the Madagascar Ministry for Water and Forests (research permits: N^0^162/13/MEF/SG/DGF/DCB.SAP/SCB and N^0^130/13/MEF/SG/DGF/DCB.SAP/SCB; export permit N^0^149N-EA08/MG14) and in strict accordance with guidelines posted by the American Veterinary Medical Association. All field protocols employed were pre-approved by the Princeton University Institutional Animal Care and Use Committee (IACUC protocol # 1926, July 2013; #1989, June 2014), and every effort was made to minimize discomfort to animals.

### 2.3 Molecular analysis

Frozen kidney tissue samples from all lethally sampled rats were transported to the Centers for Disease Control and Prevention (CDC), Fort Collins, CO. Briefly, DNA was extracted using Qiagen QIAamp tissue kits (QIAGEN, Valencia, CA, USA) according to the manufacturer’s instructions, and DNA extractions were examined for *Bartonella* spp. by conventional PCR targeting NADH dehydrogenase gamma subunit (*nuoG*) gene, which is optimal for tissue studies (Bai et al., 2015).

Ectoparasite samples collected from *R. rattus* in the District of Ankazobe were processed at the University at Buffalo (Buffalo, NY, USA), following the same procedures outlined for host tissues. DNA was extracted from a subset of samples, representing at least of one of each arthropod type recovered from an individual rat host (for a total of 24 *Synopsyllus fonquerniei,* 8 *Xenopsylla cheopsis,* 12 *Echidnophaga gallinacea,* 2 *Polyplax* sp. lice, 6 *Haemaphysalis* sp. ticks, and 6 mesostigmatid mites). Extractions were amplified for *Bartonella* spp. DNA by conventional PCR targeting the nuoG gene. Ectoparasite voucher specimens were slide-mounted and identified using available taxonomic keys.

The obtained *nuoG* sequences from both rodents and arthropod ectoparasites were clustered to the putative species level by comparing similarity with *Bartonella* spp. sequences previously deposited in GenBank (Table S1), including species identified in rats of the genus *Rattus.* Further details of our molecular processing, including primer sequences, are delineated in Text S1.

### 2.4 Quantitative analysis

All quantitative analysis was conducted in R v. 3.1 for MacIntosh (R Foundation for Statistical Computing, Vienna, Austria). Analyses followed two major themes: (1) Building on techniques from epidemiology, we first modeled age-prevalence for each of the four main species of rodent-borne *Bartonella* highlighted in our molecular analyses, allowing for age-specific variation in the force of infection and accounting for deviations across sampling sites; and (2) Focusing on the subset of our dataset for which ectoparasite samples were available, we statistically described the distribution and prevalence patterns of arthropod ectoparasites of *R. rattus* rodents in Madagascar, highlighting phylogenetic relationships across the host-parasite-vector continuum that could underpin observed patterns of infection in the host community. We here summarize our data analysis procedures in brief; the full details of our methodology are elucidated in Text S2.

#### 2.4.1 Force of infection in age-prevalence models

Explicit age determination of wild-caught *R. rattus* is virtually impossible; thus, we instead estimated age from the rodents’ weights using the von Bertalanffy equation (Ricker, 1979): *W(t)* *=* *W(1 − e^−k(t−t0)^)* where *W* is the mass of the largest individual in the dataset and *k* is a species-specific constant. In our model, *k* = 0.0207 for *R. rattus* from the literature (Rajagopalan, 1970), though see Text S3 for consideration of alternative values for *k*. The von Bertalanffy approximation is believed to provide one of the most reliable estimates of age for the Rodentia order (Griffiths and Brook, 2005, Zullinger et al., 1984). We calculated rat age (in days) separately for male and female individuals and also classed rodents into biologically-relevant 15-day age bins (i.e. infant: 0–15 days; juvenile: 16–30 days; adult: 30+ days), though see Text S3 for evaluation of the sensitivity of our results to variation in age bin duration (Fig. 1B; Yoon et al., 2014).

Using these estimates for rodent age, we followed Long et al. (2010) and Pomeroy et al. (2015); to model age-prevalence for each of the four *Bartonella* spp. and quantify the age-specific force of infection (FOI, λ), or the per capita rate at which susceptible hosts become infected. In the case of a non-immunizing persistent infection, hosts are born susceptible, and the total time of infection exposure increases with age, resulting in higher prevalence in later age classes because individuals of a given age (*a*) will have experienced a cumulative FOI throughout their lifetime. Such data will be best fit by a Susceptible-Infected (SI) model. Similar methods can be applied to estimate FOI for transient, fully immunizing infections from age*-sero*prevalence data; in such cases, seroprevalence will also increase with age, and corresponding data (not available in this study) can be fit by a Susceptible-Infected-Recovered (SIR) model. By contrast, non-immunizing, *transient* infections should demonstrate an age-prevalence profile that first increases in early age classes, reaches a peak, then declines to some lower endemic prevalence in later age classes. Older individuals will have had greater opportunity to progress through the compartments of a corresponding Susceptible-Infected-Susceptible (SIS) model, resulting in the return of many late life individuals to the susceptible class.

We used likelihood ratio tests (LRTs) to separately select amongst SI and SIS models of varying numbers of age bins for each of the four *Bartonella* spp. in our age-prevalence dataset. We employed partial profile likelihood to compute confidence intervals on each age-specific FOI estimate in the final, best fit model (Bolker, 2008). We compared each best fit model with an alternative model allowing for deviations in the age-specific FOI across the four sites investigated in this study (Ankazobe Outside, Ankazobe Inside, Ranomafana Outside, and Ranomafana Inside). Details of all model constructions, including relevant R-code, and comparison of fits for various model forms are outlined in Text S2, Tables S2 and S3.

This framework makes several assumptions: that rodents experience no heterogeneity in infection exposure, that the sensitivity of our PCR assay is perfect, and, in the case of the SI models, that infection is lifelong and infection-induced mortality negligible. Because our data are derived from two cross-sectional sampling events during the same month of two different years, we also ignore the possibility of seasonal variations in the age-specific force of infection.

#### 2.4.2 Host-parasite-vector relationships

Focusing on the subset of our data for which ectoparasite information was available (District of Ankazobe), we next sought to understand host-parasite-vector relationships that might elucidate the force of infection trends outlined above. Using representative nuoG sequences obtained from both host and ectoparasite PCR assays (see Molecular analysis, above), we constructed a maximum likelihood phylogeny highlighting nested relationships between *Bartonella* spp. genotypes recovered from rats and their arthropods. Phylogenetic alignment was performed using *Brucella abortus* as an outgroup and the default parameters in MUSCLE (Edgar, 2004). We used jModelTest 2 to identify TIM2 + I + G as the best fit model for the data (Darriba et al., 2012) and evaluated phylogenetic relationships using a GTR + G + I model with 1000 bootstrap replicates and data partitioned by codon in RAxML (Stamatakis, 2006).

We next used logistic regression to quantify the extent to which *Bartonella* spp. infection in the host rat predicted infection in the associated arthropod ectoparasite. We chose this model form over the more intuitive inverse (i.e. vector predicting infection in host) due to uncertainty over the extent to which we completely sampled the entire vector community on a given host. Because we may have missed collection of some arthropods on some hosts, we cannot accurately assess the extent to which ectoparasite infections predict host infections. We are confident in the validity of our host sampling and PCR-assay (though, see Discussion for consideration of missed mixed infections), and therefore, chose to instead evaluate host infection status as a predictor for infection status in arthropods. Because *B. phoceensis 1* and *B. rattimassiliensis 1* were each recovered from one ectoparasite only, we restricted our analysis to *B. elizabethae 1* and *2* only. Using the glmer function in the lme4 package in R (Bates et al., 2015), we related the occurrence of *B. elizabethae 1* and *2* in ectoparasite arthropods to two predictors: parasite type (5 levels: *S. fonquerniei, X. cheopsis, E. gallinacea* fleas, and *Haemaphysalis* spp. ticks) and infection status in the rat host (5 levels: *B. elizabethae 1-*positive, *B. elizabethae 2-*positive, *B. elizabethae 1-*positive, *B. phoceensis 1-*positive, *B. rattimassiliensis 1-*positive, and negative), with a random effect on rat identity to allow for recovery of multiple arthropods from the same rat host.

We also quantified host-ectoparasite interactions using a permutation test to examine whether the proportion of concordant *Bartonella* spp. infections within each host-ectoparasite pair (i.e. separately for each ectoparasite type) was greater than expected under the null hypothesis that the *Bartonella* spp. infections of a host and its ectoparasite are independent (details in Text S4). We computed a one-tailed p-value as the proportion of permutated datasets that yielded a proportion of concordance more extreme than the corresponding statistic as calculated in the unpermuted, raw data.

Finally, we explored the question of whether *Bartonella* species ranges might be limited by habitat restrictions in the vector population, with a focus on the arthropods for which sufficient data were available: the three fleas (*S. fonquerniei, X. cheopsis,* and *E. gallinacea*). We employed logistic regression to compare the occurrence of each flea species in turn against the presence of the other two flea species on the same rat host, as well as sampling site (two levels: Ankazobe Outside, Ankazobe Inside).

## 3 Results

A total of 158 *R. rattus* rats were sampled in this study, 93 of which (58.9%) were positive for some species of *Bartonella,* which we identified as belonging to five distinct genotypes (# positive; prevalence): *B. elizabethae 1* (22; 13.9%), *B. elizabethae 2* (6; 3.8%), *B. phoceensis 1* (40; 25.3%)*, B. rattimassiliensis 1* (21; 13.3%)*, B. tribocorum 1* (1; 0.01%). Because only one positive sample was recovered for *B. tribocorum 1,* the remainder of our quantiative analyses report results for the first four species of rodent-borne *Bartonella* only.

### 3.1 Force of infection in age-prevalence models

We depict rodent age estimates from each of four sampling sites in Fig. 1B, along with biologically relevant 15-day developmental age classes, by which we binned piece-wise estimates for the age-structured force of infection. Table S2 shows the full set of age-specific SI and SIS model comparisons for all four *Bartonella* spp. genotypes, and Text S2 describes the analytical process in detail, including relevant R-code. Text S3 and Table S3 provide a detailed analysis of the sensitivity of our results to variations in the age-for-weight estimates and the duration of each age bin.

**Fig. 2:**
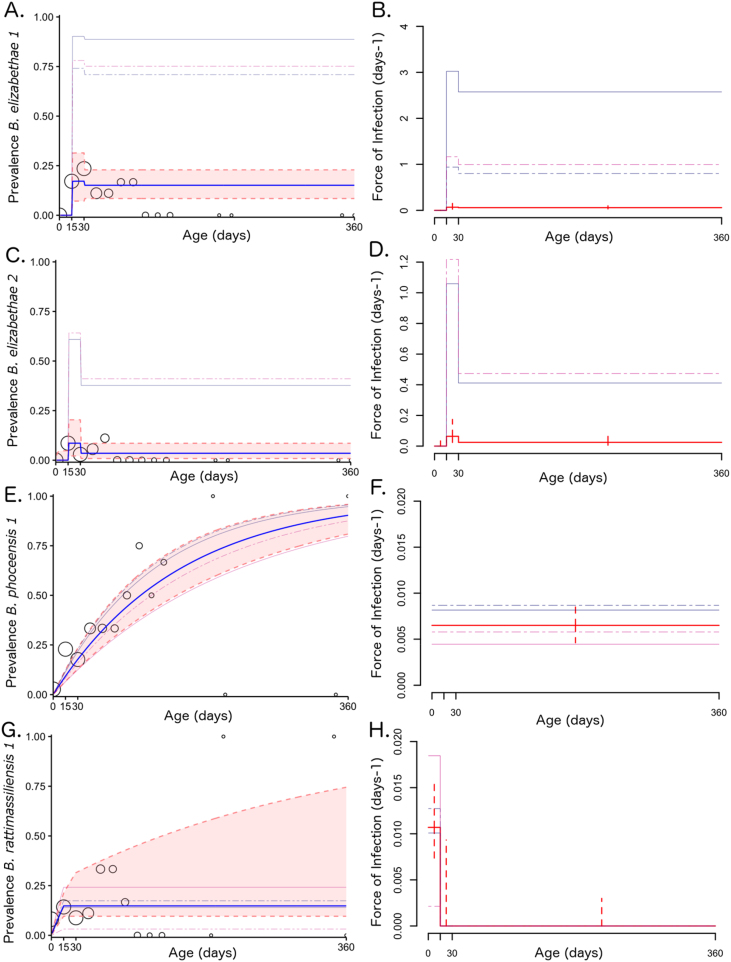
Age-prevalence and force of infection (respectively) for sampled *Rattus rattus* infected with *B. elizabethae 1* (A,B), *B. elizabethae 2* (C,D), *B. phoceensis 1* (E,F), and *B. rattimassiliensis 1* (G,H). In the age-prevalence charts (A,C,E,G), open circles signify age-stratified prevalence from the data binned over 15-day intervals, and circle size correlates to sample size within each bin. The blue line represents the expected proportion infected in each age class from the best fit model for each species (SIS with 3 age classes for *B. elizabethae 1* and *2;* SI with a constant force of infection for *B. phoceensis 1;* and SI with 3 age classes for *B. rattimassiliensis 1)*. Pink shading encompasses the 95% confidence interval as determined via partial profile likelihood, and faint background lines depict predicted prevalence from the more relaxed version of the model allowing for deviations in age-specific FOI by sampling site (navy solid = Ankazobe Outside, navy dashed = Ankazobe Inside; fuchsia solid = Ranomafana Outside; fuchsia solid = Ranomfana Inside). Confidence intervals for site-specific FOIs are listed in Table S4 (not shown in figure for ease of viewing). (For interpretation of the references to colour in this figure legend, the reader is referred to the web version of this article.)

A three-age class SIS model offered the best fit for *B. elizabethae 1* and *2* genotypes, while an SI model with constant FOI best represented the *B. phoceensis 1* data. Like *B. phoceensis 1,* the *B. rattimassiliensis 1* data were also best described by an SI model (i.e. persistent infection), though inclusion of age structure (three classes) here significantly improved the model’s ability to recover patterns in the data. Models for *B. elizabethae 1* and *2* demonstrated substantially higher estimates for FOI in the juvenile age class (15–30 day) than in both the infant (0–15 days) and post-juvenile (30+) classes, while the *B. rattimassiliensis 1* model exhibited its highest FOI in the infant class. For these three *Bartonella* species, young rodents appear to represent the age cohort supporting the majority of transmission, while *B. phoceensis 1*-infected rats experience a constant FOI across a lifespan (Fig. 2).

To explore the possibility of geographic range limits for our various *Bartonella* spp. subtypes, we compared the best fit model for each *Bartonella* genotype with a more relaxed form of the same model, which allowed for site-specific deviations in each age-class-specific estimate of the FOI (Table 1; Fig. 2). In all cases, site-specific deviations led to a significantly better fit. Because some sampling sites had zero positive cases of a particular genotype (i.e. there was no *B. elizabethae 1* recovered from Ranomafana Outside sites and no *B. elizabethae 2* recovered from Ankazobe Inside or Ranomafana Outside sites), these models were fit with only the subset of data from sites for which the pathogen was present. For this reason, we present the results for both model forms. It is possible that our more restrictive models (which do not allow for site-specific deviations in λ) violate the assumption of no heterogeneity in infection exposure if certain sampling sites are outside the habitat range for the parasite or the vector; in this case, our summary models likely *under-predict* the true FOI for *B. elizabethae 1* and *2.* If this assumption is not violated, then it is also possible that our more relaxed models allowing for site-specific deviations in λ *over-predict* the true FOI for *B. elizabethae 1* and *2* because data from low prevalence sites (zeroes in our sampling) have been excluded. More extensive field sampling of both rat hosts and ectoparasite vectors will be critical to resolving this uncertainty in the future.

**Table 1:** Comparisons of best fit FOI model per *Bartonella* spp. genotype w/& w/out sampling site deviations in λ.

*Bartonella* spp.	Model Form	Optimized Parameter Estimates								neg. log-likeli-hood	LRT^†^	p-val^††^
		Force of Infection			Site-Specific Deviations to λ				σ			
		λ (0–15)	λ (16–30)	λ (30+)	Ank. Out	Ank. In	Rano. Out	Rano.In				
*B. elizabethae 1*	SIS	1.75−23	5.93e-02	5.10e-02	–	–	–	–	0.286	52.56	–	–
	SIS (w/λ site deviations)	4.91e-35^¶^	4.83e-01^¶^	4.11e-01^¶^	6.26	1.95	–	2.42	6.58	43.98	17.16	0.00003***
*B. elizabethae 2*	SIS	4.26e-09	6.38e-02	2.46e-02	–	–	–	–	0.680	23.25	–	–
	SIS (w/λ site deviations)	4.91e-35^¶^	4.38e-01^¶^	1.70e-01^¶^	2.42	–	–	2.78	6.62	19.89	6.726	0.00950***
*B. phoceensis 1*	SI (null)	(constant FOI) 0.0065			–	–	–	–	–	75.28	–	–
	SI (null) (w/λ site deviations)	(constant FOI) 0.0067^¶^			1.22	1.29	0.662	0.862	–	73.91	2.742	0.09774*
*B. rattimassiliensis 1*	SI	1.07e-02	1.17e-12	2.71e-11	–	–	–	–	–	60.84	–	–
	SI (w/λ site deviations)	1.48e-02^¶^	7.20e-13^¶^	8.30e-12^¶^	0.681	0.860	1.247	0.145	–	57.27	7.136	0.00756***
	SIS	(constant FOI) 0.011			–	–	–	–	0.050	60.66	–	–
	SIS (w/λ site deviations)	(constant FOI) 0.515^¶^			2.04	2.28	3.64	0.306	6.47	57.46	6.394	0.01145**

^†^Likelihood ratio test ^††^associated p-value from a chi-squared distribution comparing the negative binomial log-likelihood of the more restrictive to the less restrictive model via the following equation: 2*(ll(m2)-ll(m1)) where m1 = more restrictive, no site deviation model and m2 = less restrictive, site deviation-permitting model. ^¶^These values indicate mean lambda values across subset of sites for which each model fit is tabulated; site-specific deviations are added to mean to reproduce values visualized in Fig. 2. *Statistical significance by p-value standard <0.1*, <0.05**, <0.01***.

### 3.2 Host-parasite-vector relationships

**Fig. 3:**
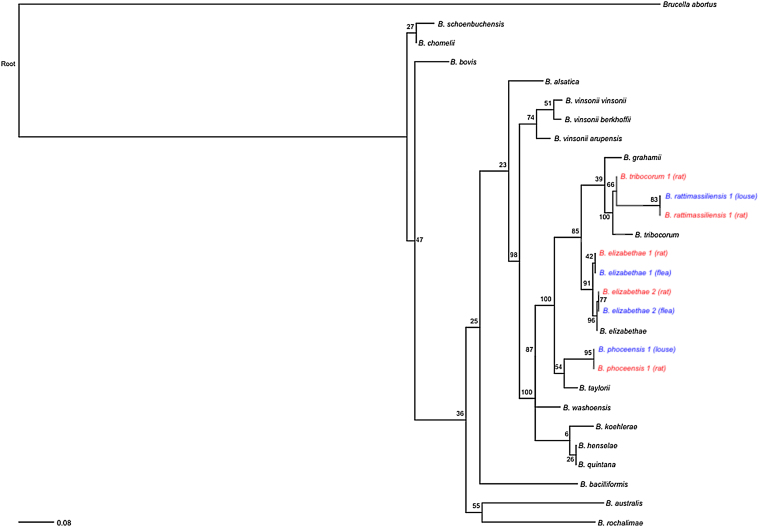
Maximum likelihood phylogeny of representative *Bartonella* genotypes obtained from nuoG gene sequencing of rodent (red) and arthropod ectoparasite (blue) samples in our dataset (outgroup: *Brucella abortus*) (RAxML, GTR + G + I model, partitioned by codon position, with 1000 bootstrap replicates) (Stamatakis, 2006). All bootstrap values are shown on corresponding nodes. Branch lengths are scaled by nucleotide substitutions per site. (For interpretation of the references to colour in this figure legend, the reader is referred to the web version of this article.)

Fig. 3 represents a maximum likelihood phylogeny of representative *Bartonella* spp. isolates recovered from nuoG sequencing of both host and arthropod ectoparasite samples. *B. elizabethae 1* and *2* sequences recovered from *S. fonquerniei* and *X. cheopsis* fleas and *B. phoceensis 1* and *B. rattimassiliensis 1* sequences recovered from *Polyplax* spp. lice nest with the same respective genotypes recovered from rodent hosts. That said, the ecological landscape of these interactions was complex and revealed considerable discordance between sequences obtained from several host-ectoparasite pairs, a pattern which has been reported in host-ectoparasite relationships for *Bartonella* spp. elsewhere (Abbot et al., 2007, Brinkerhoff et al., 2010, Tsai et al., 2010). Indeed, neither arthropod type nor host rat sequence significantly correlated with arthropod infection status in our logistic regression model (Table S5). However, our permutation tests (Text S4) demonstrated that host-ectoparasite concordance in *Bartonella* spp. for *S. fonquerniei* occurred more frequently than would be expected by chance (p = 0.007), suggestive of transmission between host-ectoparasite pairs. All cases of rat-*S. fonquerniei* concordancy were for *Bartonella elizabethae* genotypes (6/8 for *B. elizabethae 1* and 2/8 for *B. elizabethae 2*). By contrast, no host-ectoparasite concordance was observed for *X. cheopsis* (p = 1).

Table 2 summarizes the bulk of our findings across the host-parasite-vector-continuum. Most *S. fonquerniei* fleas recovered from *B. elizabethae 1* or *B. elizabethae 2*-positive rats were PCR- positive for the same *Bartonella* spp. identified in the rodent host. By contrast, none of the *S. fonquerniei* fleas recovered from *B. phoceensis 1*-positive rats were PCR-positive for the *Bartonella* spp. recovered from their hosts; notably, all of those fleas were PCR-positive for different *Bartonella* spp. than their host rat (most with *B. elizabethae 1* and one with *B. elizabethae 2*). No *X. cheopsis* recovered from *B. phoceensis 1-*positive rats were PCR-positive for the host rat *Bartonella* spp., but one tested positive for *B. elizabethae 1*. No fleas of any species recovered from *B. rattimassiliensis 1* positive-rats were positive for any sequence of *Bartonella.*

**Table 2:** Host-parasite-vector relationships from the Ankazobe subset of the data.

Sequence	Prevalence in Rattus rattus [# positive/# tested (%)]	Ectoparasites on positive rats [# positive/# tested (%)]			
		*Synopsyllus fonquerniei*	*Xenopsylla cheopis*	*Echidnophaga gallinacea*	Other
*B. elizabethae 1*	19/76 (25)	6/9 (66.7)	–	0/2 (0)	1/3 (33.3)^c^
*B. elizabethae 2*	4/76 (5.3)	2/2 (100)	–	–	–
*B. phoceensis 1*	25/76 (32.9)	0/9^a^ (0)	0/2^b^	0/3 (0)	1/4 (25)^d^
*B. rattimassiliensis 1*	11/76 (14.5)	0/2 (0)	0/1 (0)	0/2 (0)	1/2^d^

a Eight of these 9 were positive for *B. elizabethae 1* and 1 for *B. elizabethae 2* in spite of host infection.

b One of these two was positive for *B. elizabethae 1* in spite of host infection.

c The one positive “other” for *B. elizabethae 1* was a mesostigmatid mite.

d The positive ‘others’ for *B. phoceensis 1* and *B. rattimassiliensis 1* were *Polyplax* spp. lice.

Though not the focus of our sampling, we opportunistically collected two *Polyplax* spp. lice from a *B. phoceensis 1* positive rat and a *B. rattimassiliensis 1* positive rat. Both lice tested positive for the *Bartonella* genotype with which their host rats were infected. Table S6 reports raw prevalence of each *Bartonella* spp. genotype recovered from the arthropod ectoparasites assayed in our study. In addition to those arthropods already highlighted, we also sequenced several *E. gallinacea* fleas, *Haemaphysalis* spp. ticks, and mesostigmatid mites; of these, only one mite tested positive for one *Bartonella* genotype (*B. elizabethae 1),* suggesting a relatively minor role for these additional arthropods in transmission of the four *Bartonella* genotypes explored in this analysis.

**Fig. 4:**
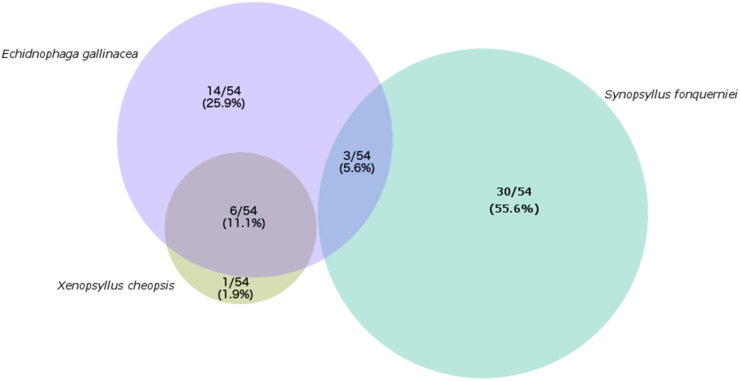
Venn Diagram of flea co-infestation for 54 *R. rattus* rats from our dataset, for which ectoparasites were isolated and identified. Numbers within each cell give the raw number and corresponding percent (%) of these 54 rats found infested with each combination of ectoparasites. Circle size scales with percentage.

Infestation with *S. fonquerniei* was significantly, negatively correlated with infestation with both *X. cheopsis* and *E. gallinacea;* in fact, no individual rats were co-infected with *S. fonquerniei* and *X. cheopsis,* though *E. gallinacea* co-infested with both other flea types (Fig. 4; Table 3). Sampling site proved to be a statistically significant predictor of flea infestation for all flea types, with “outside” captures positively correlated with infestation with *S. fonquerniei* and negatively correlated with infestation with *X. cheopsis* and *E. gallinacea; X. cheopsis* was never recovered from an outside sampling site. These findings are in congruence with previous reports of flea distributions in Madagascar (Duplantier et al., 2005). The relative absence of *S. fonquerniei* in outdoor localities and *X. cheopsis* in indoor localities in the District of Ankazobe underlines the assumptions in our FOI analyses: if a certain ectoparasite serves as the exclusive vector for one *Bartonella* spp. genotype, then its absence in certain habitats could indicate that rodent infection exposure is heterogenous by habitat type, thus validating the omission of sites lacking in data for a given pathogen in our model fits.

**Table 3:** Predictors of infestation by flea type on R. rattus, from GLM.

Flea	Predictor	Slope	Lower CI^†^	Upper CI^†^	p-val
*S. fonquerniei*	*X. cheopsis*	–*never recovered together*–			
	*E. gallinacea*	−0.896	−2.197	0.4061	0.1775
	Outside site	1.515	0.1732	2.857	0.0269**

*X. cheopsis*	*S. fonquerniei*	–*never recovered together*–			
	*E. gallinacea*	0.613	−1.816	3.042	0.621
	Outside site	*–never recovered outside–*			

*E. gallinacea*	*S. fonquerniei*	−0.896	−2.197	0.4059	0.1775
	*X. cheopsis*	1.267	−1.031	3.565	0.2799
	Outside site	−2.285	−3.555	−1.016	0.0004 ***

**^†^**Confidence intervals were computed for the slope of each interaction as slope ±1.96*standard error. * Indicates statistical significance if predictor removed from model, p-value <0.05**, <0.01***

## 4 Discussion

We here report the first account of *Bartonella* spp. infection in rodent hosts and associated arthropod ectoparasites in Madagascar. Our molecular analyses identified five distinct species of *Bartonella* (*B. elizabethae 1, B. elizabethae 2, B. phoceensis 1, B. rattimassiliensis 1,* and *B. tribocorum 1*) infecting *R. rattus* rats and/or associated arthropod ectoparasites in two disparate ecosystems of central Madagascar. We first explored the transmission dynamics of each of four *Bartonella* species for which there were sufficient data; age-structured SIS models best recovered patterns in the age-prevalence data exhibited by rats infected with *B. elizabethae 1* and *2,* while SI models provided a better fit to the data for *B. phoceensis 1* and *B. rattimassiliensis 1.* We then investigated host-parasite-vector relationships in our data, recovering nested sequences of all four *Bartonella* spp. in rat hosts and associated ectoparasites, with *B. elizabethae 1* and *2* found in *S. fonquerniei* and *X. cheopsis* fleas and *B. phoceensis 1* and *B. rattimassiliensis 1* in *Polyplax* spp. lice. These patterns are suggestive of a role for these arthropods in the transmission of respective *Bartonella* spp. In particular, concordant pairings of *B. elizabethae 1* and *2* genotypes in host-ectoparasite pairs occurred more frequently than expected by chance for *S. fonquerniei* fleas, underlining the likely importance of these arthropods in the transmission dynamics of the *B. elizabethae* complex. We demonstrated significant habitat restrictions on the range of *S. fonquerniei,* which could limit the range of the *B. elizabethae*, a known zoonotic agent, in Madagascar.

For zoonotic pathogens, the spillover force of infection is influenced by pathogen dynamics in the reservoir host, the animal-human contact rate, and the susceptibility of the spillover host (i.e. humans) to infection (Lloyd-Smith et al., 2009). Though explicit models of animal-to-human pathogen spillover are rare in the scientific literature, the few that do exist present scenarios in which spillover events are driven by epizootic peaks of infection in the reservoir population, concomitant with large-scale shedding of infectious material into the environment (Keeling and Gilligan, 2000, Sauvage et al., 2007). Consistent with these model scenarios, we found that zoonotic *Bartonella* species demonstrated higher forces of infection than genotypes not known to pose zoonotic risks. Indeed, *B. elizabethae 1* and *2* in juvenile rats demonstrated forces of infection some ten orders of magnitude higher than anything recovered for *B. rattimassiliensis 1* or *B. phoceensis 1,* thus offering a potential avenue for zoonotic transmission. Additionally, higher forces of infection are generally associated with more virulent, highly replicating pathogens, likely to cause pathogen-induced mortality and/or place greater evolutionary pressure on hosts to develop resistance; such theories are also consistent with our finding that a transmission model incorporating infection recovery (indicative of immune defense) provided the best fit to the *B. elizabethae 1* and *2* data. Intriguingly, our SIS model for *B. elizabethae 1* and *2* did not completely recapitulate the data trend of prevalence declining to zero in highest age class rodents (instead, leveling out to an equilibrium prevalence), suggesting that a Susceptible-Infected-Recovered (SIR) model form, by which pathogen clearance is immunizing, or an SI model incorporating pathogen-induced mortality might be more appropriate. Careful genotype-specific reporting of age-structured *sero*prevalence patterns against particular *Bartonella* spp. antigens would enable these inferences; previous attempts to investigate the serological landscape of rat-borne *Bartonella* spp. have yielded conflicting results (Kosoy et al., 2004a, Kosoy et al., 2004b, Kosoy et al., 1997).

Our site-specific estimates for age-specific FOI were also substantially higher for *B. elizabethae 1* and *2* than our cumulative estimates in models not allowing for location-specific deviations because sites in which the pathogen was not detected were excluded while fitting the deviation-permitting model. We are left with the question of whether our cumulative model under-predicts the force of infection for these *Bartonella* spp. by including susceptible hosts outside the habitat range of the pathogen or whether our site-deviation model over-predicts the force of infection by excluding data from low prevalence sites.

Our subsequent descriptive analyses build on our FOI analyses to offer evidence for the first hypothesis, supporting the role of *S. fonquerniei,* and, to a lesser extent, *X. cheopsis,* in the transmission of *B. elizabethae 1* and *2* among Malagasy *R. rattus* rats. The ranges of vector-borne parasites are limited by the ranges of their hosts and vectors, and ectoparasite infestations in *R. rattus* examined in our study showed distinctive signatures of habitat limitations and mutual exclusion. *S. fonquerniei* was recovered predominantly from outside sampling sites and never in concert with *X. cheopsis*, which was found exclusively on rats trapped inside households. If *S. fonquerniei* plays an essential role in the transmission cycle of *B. elizabethae*, then we might expect rats recovered from indoor sampling sites to experience a lower force of infection for *B. elizabethae* genotypes than rats trapped outside; broadly, this is the pattern exhibited in our data. Previous work demonstrates that *S. fonquerniei* is limited in geographic extent to outdoor localities above an 800 m elevation gradient in Madagascar, a factor believed to be important in predicting the range limits for another zoonotic bacterial pathogen for which *S. fonquerniei* serves as a vector: *Yersinia pestis,* the causative agent in bubonic plague.

Though ectoparasite samples were not collected from the Ranomafana region, we hypothesize that sites in this region are located at the elevation limit (∼900 m) of the highland distribution (800 m-restricted) of *S. fonquerniei.* It is possible that *S. fonquerniei* fleas and the pathogens which they support are consequently less abundant in this locale. Future studies including more categorically lowland sites will be critical to evaluating whether *B. elizabethae 1* and *2* can be independently maintained among *R. rattus* rodent hosts and *X. cheopsis* arthropods in Madagascar, or whether, as seems to be the case for plague, these *Bartonella* subtypes require the contributions of *S. fonquerniei* to their transmission cycle for persistence. Additionally, if future serological work confirms that a model incorporating parasite-induced mortality better recapitulates the dynamics of *B. elizabethae* infections in rodent hosts, then a strategy of independent pathogen maintenance in arthropod vectors (i.e. *S. fonquerniei*) may be necessary to explain *Bartonella* spp. persistence. Recent work posits a role for *Bartonella* spp. as intracellular endosymbionts of their arthropod vectors (similar to *Wolbachia* spp.) (Tsai et al., 2011); the diversity of *Bartonella* genotypes, independent of the rodent host population, exhibited amongst potential arthropod vectors in Madagascar is compatible with such a hypothesis.

Our work also recovered nested sequences of *B. phoceensis 1* and *B. rattimassiliensis 1* in *Polyplax* spp. lice and associated rodents, suggestive of a role for lice in the transmission of these other *Bartonella* species. This interaction is inconclusive due to sampling size; however, *B. phoceensis* and *B. rattimassiliensis* sequences have been recovered from louse ectoparasites of sampled rodents before (Reeves et al., 2006), while previous attempts to isolate these sequences from the fleas of infected rats have also failed (Gundi et al., 2004). Reviews of the literature thus posit a role for lice as vectors of *B. phoceensis* and *B. rattimassiliensis* (Billeter et al., 2008), a hypothesis which our data support.

Our study highlights the power of age-structured pathogen prevalence data, by which age-specific patterns in the force of infection can be inferred. We estimated rodent age from mass via the von Bertalanffy equation (Ricker, 1979), though future studies could incorporate more precise measurements of age. In particular, in lethally sampled mammals, including *Rattus* spp. rats, dried eye lens weight has been demonstrated to correlate closely with animal age (since eye lenses grow continuously throughout a mammal’s life) (Hardy et al., 1983) and could offer a more rigorous approximation of rodent age for future analyses. Additionally, future field studies should include a skeletal measure of rodent size (akin to the routine forearm measurement for bats) so that mass-per-body size relationships can be inferred. We have used rodent weight (mass) to estimate age; however, animals will vary substantially in physiological condition within a population and across a season. Among adult (75+ day) rats in particular, skeletal growth tends to cease (Yoon et al., 2014), and weight for body size may offer a better reflection of the nutritional condition of the rodent host, which can play a key role in immune mechanisms of pathogen clearance and control (Dowell, 2001).

Finally, we report the results of PCR amplification and sequence alignment of one genotype of *Bartonella* spp. for each positive rodent host and arthropod vector. Mixed infections of multiple *Bartonella* spp. in the same host and/or vector have been reported in the past (Abbot et al., 2007, Gurfield et al., 2001, La Scola et al., 2002, Rolain et al., 2003), and it is possible that our molecular analyses have preferentially amplified certain *Bartonella* genotypes over others and missed identification of coinfected individuals. In the future, more diverse sampling of multiple host tissues and/or red blood cell could help deter preferential amplification of certain genotypes. If, as exhibited in our data, more diverse tissue sampling continues to recover only one *Bartonella* spp. per rodent host, then such patterns could indicate that simultaneous infections with the multiple species of *Bartonella* present in this system are often fatal for their hosts. The extent to which coexistence of these multiple *Bartonella* spp. is mediated by inter-species competition versus landscape-level niche partitioning among parasite species with different ranges (subject to the range limits of their respective vectors) remains a question for further study.

## 5 Conclusions

Our analyses shed light on the transmission dynamics and genotype-specific host-parasite-vector associations for *Bartonella* spp. infections in *Rattus* spp. hosts and associated arthropod ectoparasites in Madagascar. We distinguish between the infection dynamics of the *B. elizabethae* complex, a known zoonotic pathogen, which appears to maintain a transient presence in its rodent hosts, and those exhibited by *B. phoceensis* and *B. rattimassiliensis*, which appear to operate as persistent infections. Many ecological studies have reported data from diverse *Bartonella* species as a single composite *Bartonella* spp. in the past (e.g. Bai et al., 2008, Janecek et al., 2012, Morway et al., 2008, Young et al., 2014). While such a practice may provide heightened statistical power, our findings suggest that this method will not reveal genotype-specific ecological associations and infection dynamics for diverse pathogens that span a broad range of host virulence strategies and differ substantially in their potential for zoonotic transmission.

## Competing interests

The authors declare that they have no competing interests.

## **Author**s’ contributions

CEB, APD, CJEM, MYK, and KD devised the study. CEB, EOY, and HCR collected field samples. YB and MYK performed molecular analyses of rodent tissue samples. HS and KD performed molecular analyses and species identifications of arthropod ectoparasites. CEB, CJM, EOY, and APD carried out the statistical analyses of the data. CEB, CJEM, APD, MYK, and KD wrote the manuscript, which all other authors read and edited.
