# Supplementary material for: Elucidating transmission dynamics and host-parasite-vector relationships for rodent-borne Bartonella spp. in Madagascar
Source: Epidemics. 2017 Sep;20:56–66. doi: 10.1016/j.epidem.2017.03.004 (PMC5608689; doi:10.1016/j.epidem.2017.03.004)
Supplement: Supplementary file 1 [file mmc1.docx]

**Supplementary Material**

**Text S1: *Materials and Methods***

***Field Sampling***

The District of Ankazobe ranges in elevation from 1300m - 1650m and can be characterized as a high altitude lichen forest with temperatures in the austral winter (the period in which these studies were conducted) ranging from 9-14°C (Vallan, 2000). Ranomafana National Park spans an elevation range from 500-1,500m and is a mountainous region of mixed lowland rainforest, cloud forest, and high plateau forest, with winter temperatures ranging from 11-17°C (Gerber et al., 2010). Our trapping sites in Ranomafana were located near the Centre ValBio Research Station and the village of Vohiparara, spanning an elevation gradient of approximately 900-1200m.

*Rattus rattus* were trapped in both localities using Sherman live traps (3x3.5x9”) baited with a combination of oats, peanuts, and crushed bananas. Traps were set at dusk and checked the following dawn (in the case of Ankazobe) or three hours after setting (in the case of Ranomafana), then left closed during the day. No traps were set during heavy rains or winds to avoid exposing animals to cold temperatures. Native rodents were ear-marked with a felt-tip pen and released, while *Rattus rattus* were brought back to a field lab for processing. In total, 158 *R. rattus* were anesthetized with a ketamine – xylazine concoction, then humanely dispatched via cervical dislocation. In the District of Ankazobe, *R. rattus* were placed in sealed Ziploc bags with 5ml of 97% ethanol residue for 10min directly following euthanasia; ectoparasites were then collected from the bag and combed from the rodent’s body into vials with ethanol for storage and transport. Ectoparasites were not collected by researchers at Ranomafana National Park; as such, data from Ranomafana sites are not included in all host-parasite-vector analyses presented here. Finally, all euthanized *R. rattus* were harvested for kidney samples which were frozen in cryogenic tubes deposited in liquid nitrogen (in the case of Ankazobe) or transported to a -80°C freezer at Centre ValBio Research Station (in the case of Ranomafana). An additional 30 *Mus musculus* were captured, euthanized, and harvested for kidneys from Ranomafana sites and included in the *Bartonella* spp. assays presented here. All samples were eventually transported to -80°C freezers at Institut Pasteur de Madagascar to await export to the US for molecular assay.

***Molecular analysis***

DNA was extracted from frozen kidney tissues of all sampled rats (158) and mice (30), as well as from ectoparasite arthropods recovered from these same rats, using Qiagen QIAamp tissue kits (QIAGEN, Valencia, CA, USA) according to the manufacturer’s instructions. As outlined in the main text. DNA extractions were examined for *Bartonella* spp. by conventional PCR targeting NADH dehydrogenase gamma subunit (*nuoG*) gene, which is optimal for tissue studies (40). PCR was carried out in a PTC-200 Peltier Thermal Cycler using the following primers: nuoG_F (5’- GGC GTG ATT GTT CTC GTT A – 3’) and nuoG_R (5’-CAC GAC CAC GGC TAT CAA T-3’). PCR products were visualized on a 1.5% agarose gel after electrophoresis using ethidium bromide staining.

No kidney tissues from the 30 *M. musculus* individuals sampled in the Ranomafana region were positive for any *Bartonella* spp. Thus, all analysis of host-parasite relationships are in reference to *R. rattus* samples only.

Positive PCR products were purified using a QIAquick PCR Purification Kit (Qiagen) and sequenced with a 3130 Genetic Analyzer (Applied Biosystems, Foster City, CA, USA). Phylogenetic analysis was performed using the CLUSTAL W program within MegAlign of the Lasergene software package (DNAstar, Madison, WI, version 12). The obtained *nuoG* sequences from both rodents and arthropod ectoparasites were then clustered to the putative species level by comparing similarity with *Bartonella* spp. sequences previously deposited in GenBank (Table S1).

**Text S2*: Force of Infection Analysis***

***Theory***

We considered two different forms of age-prevalence models in our estimation of the age-specific force of infection (λ). The data examined were PCR-sequences for four distinct *Bartonella* spp. lineages (*B. elizabethae 1, B. elizabethae 2, B. phoceensis 1,* and *B. rattimassiliensis 1*). Separately, for each *Bartonella* spp., hosts were classified as either (0) infected or (1) uninfected. Age was estimated from rodent weight separately for male and female rodents via the von Bertalanffy equation (Ricker, 1979): *W(t) = W(1-e^-k(t-t0)^)* in which *W* represents the highest weight class rodent in the data subset; for males, this was a rodent of 175 g and for females, this was a rodent of 152 g. In our model, k = 0.0207 for *R. rattus* from the literature (Rajagopalan, 1970). See Text S3 and Figure S2 for analysis of the sensitivity of our results to changes in k and alterations in the age class bin size.

Following Long et al., 2010, we organized our data into individual datasets for each of our four *Bartonella* spp. genotypes such that each captured rat was represented by a unique line, with the column ‘age’ listing the age of the individual and the column ‘sick’ listing a 0 for uninfected individuals (for the particular genotype in question) and a 1 for infected individuals. Additionally, to enable the sample site deviations allowed in our later model forms, we included a column ‘site’ which listed the four sample sites from which our data were gathered: Ankazobe Outside, Ankazobe Inside, Ranomafana Outside, and Ranomafana Inside.

*SI Model*

Following Pomeroy et al., 2015, we first consider a two disease state system in which rodent hosts are classified into Susceptible and Infected proportions of the total population, such that S + I = 1.

$$\lambda$$

S

I

In a traditional SIR model, the transmission rate is typically represented as β, meaning that susceptible individuals progress from S to I at a rate βSI. We here represent this transmission with the force of infection, where $\lambda=\beta I$ which better captures the time (or age)-varying trajectory of infection. Assuming an age-specific force of infection, represented by $\lambda\left( a \right)$, it then follows that:

$$\frac{dI(a)}{da}=\lambda(a)\left( 1-I\left( a \right) \right)$$

Solving for $I(a)$ gives:

$$I\left( a \right)=1+C*exp\left[ -\int_{0}^{a} \lambda(a)da \right]$$

We then make the assumption that all individuals are susceptible at birth, such that $I\left( 0 \right)=0$, by which $C=-1$. Incorporating this assumption into the previous equation, we find that:

$$I\left( a \right)=1-exp\left[ -\int_{0}^{a} \lambda\left( a \right)da \right]$$

where λ(a) is the age-dependent force of infection. Following Long et al., 2010, we assume a constant FOI for pre-determined intervals based on biologically relevant 15-day age classes in rodent ontogeny. When the FOI is assumed to be piece-wise constant across k age classes and each segment has a starting age $l_{a}$ and duration $d_{k}$, the integrand in the preceding equation for an individual of an age within the *k*’th age class is given by:

$$\int_{0}^{a} \lambda\left( a \right)da=\lambda_{k}\left( a-l_{k} \right)+\sum_{a=0}^{k-1} \lambda_{a}d_{a}$$

Adopting R-code from the supplementary material of Long et al., 2010, we can estimate the age-class specific forces of infection $\lambda\left( a \right)$ by minimizing the negative log-likelihood with the R function dbinom(). We first write a general function to return the negative log-likelihood from the model when compared with the data. The function inputs are a series of starting values for the $\lambda\left( a \right)$estimates (par), the dataset to be analyzed (data) and the lower cut-off for each age class in our model (cate). The function then follows as:

loglikpc=function(par,data, cate){

dur=c(diff(cate), 0)

ll=0

for(a in 1:length(data$age)){

dummy1=data$age[a]>cate

dummy2 = data$age[a]>cate & !c(data$age[a]>cate[-1], FALSE) dummy1=c(data$age[a]>cate, FALSE)[-1]

inte=sum(dur*exp(par)*dummy1)+ exp(par[dummy2])*(data$age[a]-cate[dummy2])

p=1-exp(-inte)

ll=ll+dbinom(data$sick[a],1,p,log=T)

}

return(-ll)

}

As described by Long et al., 2010, the first line of this function calculates the duration of each age class, the second line sets the log-likelihood to zero, and lines 3-9 encompass a for-loop which calculates the hazard of infection for each individual in the dataset by integrating over the age classes already passed and the duration of time already spent in the current age class. Dummy variables are used to identify the preceding and current age class for each individual of age *a*. Line 9 gives the predicted hazard of infection for a given age (*a*) from the catalytic model, and line 10 evaluates the observed infection status for an individual of this age against the prediction using the binomial log-likelihood.

We can then estimate $\lambda\left( a \right)$ using the optim() function in R. For this section of the analysis, we write a separate wrapper function, which brings in a vector of cut-off ages (i.e. cate=c(0,15,30) for *B. elizabethae 1* and *2* and *B. rattimassiliensis 1* in our analysis), provides some reasonable starting estimates for age-specific $\lambda$, and then optimizes $\lambda(a)$ using the quasi-Newton ‘BFGS’ method recommended in Bolker, 2008. The optimization function thus takes the form (here for the *B elizabethae 1* subset of our data):

out = optim(par=log(c(4e-15,4e-15,4e-15), fn=loglikpc, cate= c(0,15,30), method="BFGS", data=data.eliza1, control=list(trace=2, maxit=1000))

The optimized age-specific force of infection estimates are then given by out$par, while the negative log-likelihood of the comparison of the fit of our dataset against the age-prevalence predicted by the SI model with these parameters is given by out$value.

Separately, for all four *Bartonella* spp. genotypes examined in our analysis, we compared fits of the above SI model under single (null), three, and six age class scenarios. See Table S2 for the full set of results from the likelihood ratio tests, which we used to compare these models. The three-age class model fit best for *B. elizabethae 1* and *2* and for *B. rattimassiliensis 1,* while the null model provided the best description of the data for *B. phoceensis 1.* Because the likelihood ratio tests between the null and three age class fits were insignificant for *B. rattimassiliensis 1* (LRT = 0.38, p= 0.538), we also display the prevalence and force of infection predicted by the null model in Fig S1.

*SIS Model*

Because our SI model form was unable to recapitulate the trend of declining prevalence at higher age class exhibited in our *B. elizabethae 1* and *B. elizabethae 2* data, we compared this model against a Susceptible-Infected-Susceptible form, by which individuals were allowed to clear infections and return to the susceptible class.

As before, now following Pomeroy et al., 2015, we still consider a two disease state system in which rodent hosts are classified into Susceptible and Infected proportions of the total population, such that S + I = 1. However, our model now allows for recovery from infection:

$$\lambda$$

$$\sigma$$

S

I

|  |
| --- |

Assuming an age-specific force of infection, represented by $\lambda\left( a \right)$, and a constant rate of recovery $(\sigma$), it then follows that:

$$\frac{dI(a)}{da}=\lambda\left( a \right)\left( 1-I\left( a \right) \right)-\sigma*I\left( a \right)$$

Solving for $I(a)$ then gives:

$$I\left( a \right)=\frac{\lambda(a)}{\lambda\left( a \right)+\sigma}+C*exp\left[ -\int_{0}^{a} \left( \lambda\left( a \right)+ \sigma\right)da \right]$$

As before, we assume that all individuals are susceptible at birth such that $I\left( 0 \right)=0$, by which we solve for $C=-\frac{\lambda(a)}{\lambda\left( a \right)+\sigma}$ and then establish the following:

$$I\left( a \right)=\frac{\lambda(a)}{\lambda\left( a \right)+ \sigma}\left( 1-exp\left[ -\int_{0}^{a} \left( \lambda\left( a \right)+ \sigma\right)da \right] \right)$$

Still considering $\lambda(a)$ as a piece-wise constant across each age bin, we now represent the integral from the preceding equation as:

$$\int_{0}^{a} \left( \lambda\left( a \right)+ \sigma\right)da=\lambda_{k}\left( a-l_{k} \right)+\sum_{a=0}^{k-1} \lambda_{a}d_{a}+ \sigma\left( a-l_{k} \right)+\sum_{a=0}^{k-1} \sigma d_{a}$$

which reduces to:

$$\int_{0}^{a} \left( \lambda\left( a \right)+ \sigma\right)da=\lambda_{k}\left( a-l_{k} \right)+\sum_{a=0}^{k-1} \lambda_{a}d_{a}+ \sigma a$$

We then adjust our log-likelihood function accordingly and estimate parameters in the same way as described above. The new log-likelihood function, including recovery from infection is given by:

loglikpc=function(par,data, cate){

dur=c(diff(cate), 0)

ll=0

for(a in 1:length(data$age)){

dummy1=data$age[a]>cate

dummy2 = data$age[a]>cate & !c(data$age[a]>cate[-1], FALSE) dummy1=c(data$age[a]>cate, FALSE)[-1]

inte=sum(dur*exp(par)*dummy1)+ exp(par[dummy2])*(data$age[a]-cate[dummy2]) + sum(dur*par[length(par)]*dummy1) + (par[length(par)])*(data$age[a]-cate[dummy2])

discount_lambda = exp(par[dummy2])/(exp(par[dummy2]) + par[length(par)])

p= discount_lambda*(1-exp(-inte))

ll=ll+dbinom(data$sick[a],1,p,log=T)

}

return(-ll)

}

where par[length(par)] signifies the $\sigma$ (pathogen clearance) parameter. When offering the function initial estimates for each parameter, these estimates must be ordered first with the age-specific initial values for $\lambda$ and with the initial guess for $\sigma$ last.

*Site-Specific Deviations in FOI*

After choosing the best fit model in terms of age class (one, three, or six) and form (SI vs. SIS), we allowed for sample site-specific deviations in our optim()-estimated values for the age-specific force of infection $\lambda\left( a \right)$. We adapted both the SI and SIS models code to allow for this possibility. We illustrate these model alterations in the SI log-likelihood function exhibited here:

loglikpc.site=function(par,data, cate, reg.vect){

dur=c(diff(cate), 0)

ll=0

for(a in 1:length(data$age)){

dummy1=data$age[a]>cate

dummy2 = data$age[a]>cate & !c(data$age[a]>cate[-1], FALSE)

dummy2 = c(dummy2, rep(FALSE, length(reg.vect)))

dummy1=c(data$age[a]>cate, FALSE)[-1]

dummy1=c(dummy1, rep(FALSE, length(reg.vect)))

dummy3 = c(data$site[a]==reg.vect)

dummy3 =c(rep(FALSE,length(cate)), dummy3)

inte=sum(dur*exp( (par) + (par[dummy3]))*dummy1) + (exp(par[dummy2]) + (par[dummy3])))*(data$age[a]-cate[dummy2])

p=1-exp(-inte)

ll=ll+dbinom(data$sick[a],1,p,log=T)

}

return(-ll)

}

where the input vector reg.vect is a list of the sample site names exhibited in the “site” column of our dataset. The third dummy variable now indicates which sampling site is attributable to each individual in the dataset; note that while optimizing the parameters in this function, initial parameter inputs should be ordered as a single vector *par* with all $\lambda\left( a \right)$estimates listed first, followed by a single deviation parameter for each sample site, and, finally, in the case of the SIS form (not shown), followed by the $\sigma$ (pathogen clearance) parameter. It is important to note that this function will only accept inputs for which data are available for each specified sampling site; thus, in our analyses, it was necessary to restrict the data to a subset of sites for which at least one positive sample was recovered for the genotype in question (this excluded Ranomafana Outside for *B. elizabethae 1* and both Ankazobe Inside and Ranomafana Outside for *B. elizabethae 2*).

*Establishing Confidence Intervals*

Again following Long et al., 2010, we used partial profile likelihoods to erect confidence intervals for each age-specific estimates for $\lambda$ in our analysis. To do this, we profiled the likelihood for each age-class-specific force of infection separately, minimizing the negative log-likelihood while optimizing $\lambda$ for all other age classes. In the case of *B. elizabethae 1* and *B. elizabethae 2,* which were best fit by an SIS model, we computed these confidence intervals while holding $\sigma$, the recovery rate, constant at the value optimized when all parameters were allowed to vary simultaneously (see Table 1 in the main text for values). Similarly, to compute confidence intervals for the site-specific $\lambda$ estimates depicted as faint background lines in Fig 2, we profiled each age-specific FOI estimate while fixing sigma (if applicable) and all site-specific deviations at values reported in Table 1 of the main text; other FOI parameters were optimized while we profiled over a range of values for the $\lambda$ of interest. Our R-code for this step of the analysis is identical to that displayed in the supplementary material of Long et al., 2010, but we nonetheless repeat the code here (for the SI version of the general model not allowing for site deviations only).

We modified our likelihood function to flag the segment to be profiled (which) and what value to consider for that segment (wval). The modified function is then given by the following:

loglikpc2=function(par,data, cate, which, wval){

dur=c(diff(cate), 0)

ll=0

for(a in 1:length(data$age)){

dummy1=data$age[a]>cate

dummy2 = data$age[a]>cate & !c(data$age[a]>cate[-1], FALSE) dummy1=c(data$age[a]>cate, FALSE)[-1]

par[which]=wval

inte=sum(dur*exp(par)*dummy1)+ exp(par[dummy2])*(data$age[a]-cate[dummy2]) + sum(dur*par[length(par)]*dummy1) + (par[length(par)])*(data$age[a]-cate[dummy2])

discount_lambda = exp(par[dummy2])/(exp(par[dummy2]) + par[length(par)])

p= discount_lambda*(1-exp(-inte))

ll=ll+dbinom(data$sick[a],1,p,log=T)

}

return(-ll)

}

Still following Long et al., 2010, we considered a range of candidate values for the parameter we profiled (here, $\lambda$ for age class 1 in a *B. rattimassiliensis 1* infection):

wval <- seq(-10,0, by=.1)

and created a vector of corresponding length to hold the likelihood values for each of those candidate values:

ll1 <- rep(NA, length (wval))

We then for-looped through these values, for parameter 1, while optimizing the other log-lambda estimates in our model:

for (i in 1:length(wval)){

out <- optim(par= log(c(4e-15,4e-15,4e-15)), fn=loglikpc2, cate=c(0,15,30), method="BFGS", data=data.rat1, control=list(maxit=1000), hessian=T, which=1, wval=wval[i])

}

ll1[i] <- out$value

}

ll1 <- c(na.omit(ll1))

Still borrowing code from Long et al., 2010, we used a smoothing spline to interpolate the partial likelihood profile:

tmp2=smooth.spline(wval,ll1)

new=seq(-10,0, by=.001)

interp= predict(tmp2, new)$y

and capitalized on the fact that the profile likelihood is χ2-distributed to erect 95% confidence intervals:

mle1=new[which.min(interp)]

tmp3=(predict(tmp2, new)$y-min(predict(tmp2, new)$y))-qchisq(0.95,1)

conf.int = range(new[tmp3<0])

We then repeated this analysis over a range of values for the FOI estimates for age classes two (which=2) and three (which=3).

**Text S3: *Sensitivity to Age-Weight Relationships and Age Binning***

Our final manuscript reports results based on a *k* coefficient of 0.0207 for the von Bertalanffy age-weight equation (Ricker, 1979): *W(t) = W(1-e^-k(t-t0)^).* This parameter constant is the only reported value of *k* for *R. rattus* in the literature and is based on one study in captive rats from 1970 (Rajagopalan, 1970). Zullinger et al., 1984 report values of *k* for the entire *Rattus* genus, which range from 0.0077 to 0.0355. Higher values of *k* indicate faster maturation rates, such that the model estimates rodents of larger mass to be younger than our standard *k* indicates. Lower values of *k* yield slower maturation rates, meaning that the model estimates rodents of a given mass to be considerably older than as with our standard k. We explored the sensitivity of our age determination for rodents across four values of *k*, which span the range of those previously reported in the *Rattus* genus (*k*=.001, *k*=.01, *k*=.0207, *k*=.05); we illustrate these results in Figure S2.

The lowest tested value for *k* (0.001) yielded a maximum age-from-weight estimate of 7467.4 days (20.5 years) for the largest rodent in our dataset, while the highest tested *k*-value (.05) yielded a maximum age estimate of 149.3 days (.41 years) for that same rodent. Both these values are unreasonable age estimates (respectively, too high and too low) for wild *R. rattus*; thus, we focused all subsequent analyses on comparing the literature-derived *k*=.0207, (which yielded a maximum rodent age estimate of 360.7 days, or 1 year, within our dataset) with a range of *k-*values increasing in .001 increments and spanning from *k*=.01 (which yields a maximum age estimate of 746.7 days) to .025 (which yields a maximum age estimate of 298.7 days in our dataset).

To explore the impact of variation across this range of *k* on our model fits, we wrote a wrapper function to tabulate age-from-weight using *k* for each rodent in the dataset, compute the age-prevalence values over a series of age bins and feed these data into the likelihood fitting scripts outlined above. Literature derived from lab rat studies suggests that major developmental changes in the genus *Rattus* take place on the order of every 15 to 30 days post-gestation (Yoon et al., 2014). Thus, we minimized the negative log-likelihood across our range of *k-*values under four distinct scenarios: with age bins spanning 15, 20, 25, and 30-day durations. We then selected the k-parameter that minimized the log-likelihood across our range in each of these scenarios and conducted log-likelihood ratio tests comparing this fit against that of the literature-established .0207 value for *k.* For the 15- and 20-day age bins, no value for *k* offered a significantly better fit to the data than the standard (for all four *Bartonella* spp. considered). For the 25- and 30-day age bin scenarios, *k-*values lower than the standard occasionally offered better model fits. This finding is not surprising—life history theory leads us to expect that slower maturation rates (i.e. lower values for *k*) should correspond to developmental classes of longer duration.

We then computed additional likelihood ratio tests to compare our optimized models for each age bin duration against one another. We report the results of these comparisons for all four *Bartonella* spp. of interest in Table S3. The log-likelihood was minimized with age bins of length 15 days; thus, we adopted this binning as standard across all our analyses. Estimated values for age-class specific lambdas varied with changes in age bin duration while optimizing to recover the age-prevalence patterns witnessed in the data; however, the qualitative pattern of low FOI in the earliest age class, peak FOI in the second age class, and low (close to zero) FOI again in adult age class rats for *B. elizabethae 1* and *2* and for *B. rattimassiliensis* was consistent across all binning scenarios (*B. phoceensis 1* data were best fit by a model of constant FOI). As such, we are confident in our inferences about the age-specific pattern in the relative force of infection amongst age classes in this population, but we caution against any comparisons of the raw reported value for FOI (which is sensitive to age binning) with any other systems.

**Text S4: *Permutation Test***

To explore the extent to which a *Bartonella* species recovered in a host rat was concordant with that recovered from the ectoparasite arthropod, we followed the following protocol to conduct each permutation test: We first structured our dataset allowing a separate row for every ectoparasite recovered from every host rat in the sampling. Column headers were given as: rat_seq, parasite_seq, parasite_type.

We then computed the proportion of concordant and discordant combinations for *Bartonella* species recovered in ectoparasites and corresponding rat hosts, running the analysis separately for each arthropod type and excluding entries for which no sequence was retrieved from the ectoparasite. We limited our analysis to *S. fonquerniei* and *X. cheopsis* fleas, the only ectoparasites for which sufficient data were available. Code is shown here for *S. fonquerniei:*

syn.concordants <- as.numeric(length(dat$rat_seq[dat$parasite_type=="synopsyllus" & dat$rat_seq == dat$parasite_seq & dat$parasite_seq!="negative"]))

syn.discordants <- as.numeric(length(dat$rat_seq[dat$parasite_type=="synopsyllus" & dat$rat_seq != dat$parasite_seq & dat$parasite_seq!="negative"]))

syn.tot <- syn.concordants + syn.discordants

syn.prop <- c((syn.concordants/syn.tot), (syn.discordants/syn.tot))

We then permuted our host *Bartonella* spp. 999 times without replacement using the following function, which returned the proportion of concordant/discordant pairs in each sample permutation:

permute.data<- function(data, parasite_type){

permute <- sample(data$rat_seq, replace=FALSE)

data$rat_seq <- permute

concordants <- as.numeric(length(data$rat_seq[data$parasite_type== parasite_type & data$rat_seq == data$parasite_seq & data$parasite_seq!="negative"]))

discordants <- as.numeric(length(data$rat_seq[data$parasite_type==parasite_type & data$rat_seq != data$parasite_seq & data$parasite_seq!="negative"]))

tot <- concordants + discordants

prop <- c((concordants/tot), (discordants/tot))

return(prop)

}

We then pooled our observed proportions with the proportions outputted from the permuted data. Because we hypothesized that host-ectoparasite pairs should have concordant *Bartonella* spp., we computed the p-value of the distribution of permuted and observed data as the proportion of frequencies equal to or greater than the frequency witness in our observed data.

The distribution of permutations and associated p-values (region highlighted in red) for both *S. fonquerniei* (p = .007) and *X. cheopsis* (p=1) are depicted below. The frequency of concordance in the observed data are represented by vertical red lines:


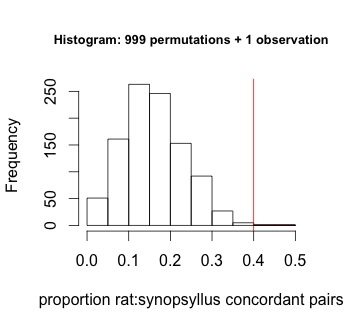

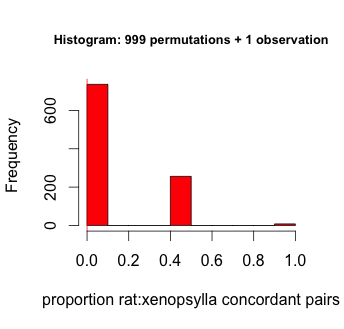


**Supplementary Tables**

**Table S1. GenBank accession numbers for *nuoG* sequences**

| **Species** | ***nuoG*** |
| --- | --- |
| *B. taylorii* | EF659943.1 |
| *B. elizabethae* | EF659940.1 |
| *B. tribocorum* | AM260525.1 |
| *B. grahamii* | EF659941.1 |
| *B. alsatica* | EF659935.1 |
| *B. vinsonii* subsp. *berkhoffii* | EF659937.1 |
| *B. vinsonii* subsp. *vinsonii* | EF659944.1 |
| *B. vinsonii* subsp. *arupensis* | EF659936.1 |
| *B. washoensis* | EF659945.1 |
| *B. henselae* | KP822813.1 |
| *B. koehlerae* | EF659942.1 |
| *B. quintana* | BX897700.1 |
| *B. rochalimae* | FN645459.1 |
| *B. schoenbuchensis* | FN645509.1 |
| *B. chomelii* | KM215699.1\| |
| *B. bovis* | EF659938.1 |
| *B. bacilliformis* | NC_008783.1 |
| *B. australis* | CP003123.1 |

**Table S2. FOI model comparisons for differing age structures in SI and SIS forms**

| ***Bartonella* spp.** | **Model Form** | **# Age Classes for λ** | **Neg. log likelihood** | **LRT^†^** | **p-value^††^** |
| --- | --- | --- | --- | --- | --- |
| *B. elizabethae 1* | SI (null) | One (null) | 62.13 | -- | -- |
|  | SIS | One | 55.64 | 12.99 | 0.00031*** |
|  | SI | Three | 55.77 | 12.73 | 0.00036*** |
|  | **SIS**^¶^ | **Three** | **52.56** | **8.08** | **0.0113**** |
|  | SI | Six | 55.77 | 0 | 1 |
|  | SIS | Six | 159.63 | -207.72 | 0.0000*** |
| *B. elizabethae 2* | SI (null) | One (null) | 27.18 | -- | -- |
|  | SIS | One | 24.96 | 4.45 | 0.035** |
|  | SI | Three | 24.82 | 4.72 | 0.030** |
|  | **SIS**^¶^ | **Three** | **23.25** | **3.14** | **.076*** |
|  | SI | Six | 24.82 | 0 | 1 |
|  | SIS | Six | 21.37 | 6.90 | 0.009*** |
| *B. phoceensis 1* | **SI (null) ^¶^** | **One (null)** | **75.28** | **--** | **--** |
|  | SIS | One | 74.23 | 2.10 | 0.147 |
|  | SI | Three | 75.98 | -1.39 | 0.238 |
|  | SIS | Three | 77.92 | -3.89 | 0.048** |
|  | SI | Six | 81.59 | -11.22 | 0.0008*** |
|  | SIS | Six | 132.52 | -101.86 | 0.0000*** |
| *B. rattimassiliensis 1* | SI (null) | One (null) | 63.22 | -- | -- |
|  | **SIS^¶^** | **One** | **60.66** | **4.91** | **0.024**^†††^** |
|  | **SI^¶^** | **Three** | **60.85** | **4.74** | **0.029**^†††^** |
|  | *Three age class-SI vs. One age class-SIS:* | | | -0.38 | 0.538 |
|  | SIS | Three | 60.69 | 0.316 | 0.574 |
|  | SI | Six | 60.85 | 0 | 1 |
|  | SIS | Six | 142.99 | -164.29 | 0.0000*** |
| ^†^Likelihood ratio test & ^††^associated p-value from a chi-squared distribution comparing the binomial log-likelihood of the more restrictive to the less restrictive model via the following equation: 2*(ll(m2)-ll(m1)) where m1 = more restrictive, fewer age class model and m2 = less restrictive, more age class model. All LRTs of an SI model compare the model highlighted with the SI model of fewer age classes (two rows above), while all LRTs of an SIS model compare the model highlighted with the SI model of the same number of age classes (directly one row above). Note that when the LRT is negative, it indicates a better fit for the more restrictive model. *Statistical significance by p-value standard <.1*, <.05**, <.01***. ^¶^ Bold highlights the best fit model per *Bartonella* spp. category as concluded by performing pairwise model comparisons between model versions with increasing complexity in number of age classes, then comparing SI versus SIS forms of the best fit number of age classes. **^†††^**Because our protocol yielded significant p-values for both the models highlighted here, we compared the two forms against one another via LRT; models were indistinguishable (LRT= 0.39; p-value=0.538). As such, we present the 3-age-class SI model in the main text (Fig 2) but also present the one age class SIS model in Fig S1. | | | | | |

**Table S3. FOI model comparisons for variation in *k-*derived age estimates and the age bin duration**

| ***Bartonella* spp.** | | **Model Form** | **k-value** | **Age Class Duration** | **Neg. log likelihood** | **LRT^†^** | **p-value^††^** |
| --- | --- | --- | --- | --- | --- | --- | --- |
| *B. elizabethae 1* | SIS  three age classes | | **.0207** | **15** | **52.56** | **--** | **--** |
|  |  |  | .0207 | 20 | 52.69 | -.26 | .610 |
|  |  |  | .017 | 25 | 52.14 | .84 | .359 |
|  |  |  | .011 | 30 | 52.33 | .46 | .498 |
| *B. elizabethae 2* | | SIS  three age classes | **.0207** | **15** | **23.25** | **--** | **--** |
|  |  |  | .0207 | 20 | 24.96 | -3.42 | .064 |
|  |  |  | .012 | 25 | 23.30 | -.10 | .752 |
|  |  |  | .0207 | 30 | 24.29 | -2.08 | .149 |
| *B. phoceensis 1* | | SI  null, one age class | .0207 | 15 | **75.28** | **--** | **--** |
|  |  |  | .0207 | 20 | 75.28 | 0 | 1 |
|  |  |  | 0207 | 25 | 60.92 | 0 | 1 |
|  |  |  | .0207 | 30 | 61.07 | 0 | 1 |
| *B. rattimassiliensis 1* | | SI  three age classes | .0207 | 15 | **60.84** | **--** | **--** |
|  |  |  | .0207 | 20 | 60.80 | .08 | .778 |
|  |  |  | 0207 | 25 | 60.92 | -.16 | .689 |
|  |  |  | .0207 | 30 | 61.07 | -.46 | .498 |
| ^†^Likelihood ratio test & ^††^associated p-value from a chi-squared distribution comparing the binomial log-likelihood of the more restrictive to the less restrictive model via the following equation: 2*(ll(m2)-ll(m1)) where m1 = the model fit with the standard k-value (.0207) and the minimum duration for each age class (15-day). When LRT results are negative, this indicates that the standard k model provided a *better* fit to the data than the alternative. | | | | | | | |

| **Table S4. Site-specific FOI confidence intervals** (not shown in Fig 2 for ease of viewing) | | | | | | | |
| --- | --- | --- | --- | --- | --- | --- | --- |
| ***Bartonella* spp.** | **Site** | **Lower CI** | | | **Upper CI** | | |
|  |  | **λ (0-15)** | **λ (16-30)** | **λ (30 +)** | **λ (0-15)** | **λ (16-30)** | **λ (30 +)** |
| ***B. elizabethae 1*** | Ankazobe Outside | 0 | 1.067 | 1.329 | 0.0021 | 7.311 | 4.447 |
|  | Ankazobe Inside | 0 | 0.331 | 0.413 | 0.0006 | 2.271 | 1.382 |
|  | Ranomafana Inside | 0 | 0.411 | 0.513 | 0.0008 | 2.819 | 1.715 |
| ***B. elizabethae 2*** | Ankazobe Outside | 0 | 0.246 | 0.098 | 0.0014 | 3.044 | 1.093 |
|  | Ranomafana Inside | 0 | 0.283 | 0.113 | 0.0016 | 3.501 | 1.257 |
| ***B. phoceensis 1*** | Ankazobe Outside | (constant FOI) 0.0071 | | | (constant FOI) 0.0088 | | |
|  | Ankazobe Inside | (constant FOI) 0.0075 | | | (constant FOI) 0.0094 | | |
|  | Ranomafana Outside | (constant FOI) 0.0038 | | | (constant FOI) 0.0048 | | |
|  | Ranomafana Inside | (constant FOI) 0.0050 | | | (constant FOI) 0.0063 | | |
| ***B. rattimassiliensis 1***  **(SI w/ age structure)** | Ankazobe Outside | 0.0082 | 0 | 0 | 0.0135 | 0.0085 | 0.0023 |
|  | Ankazobe Inside | 0.0103 | 0 | 0 | 0.0171 | 0.0108 | 0.0029 |
|  | Ranomafana Outside | 0.0150 | 0 | 0 | 0.0250 | 0.0156 | 0.0043 |
|  | Ranomafana Inside | 0.0017 | 0 | 0 | 0.0029 | 0.0018 | 0.0005 |
| ***B. rattimassiliensis 1***  **(SIS w/no age structure)** | Ankazobe Outside | (constant FOI) 0.6270 | | | (constant FOI) 1.306 | | |
|  | Ankazobe Inside | (constant FOI) 0.7002 | | | (constant FOI) 1.459 | | |
|  | Ranomafana Outside | (constant FOI) 1.118 | | | (constant FOI) 2.330 | | |
|  | Ranomafana Inside | (constant FOI) 0.0939 | | | (constant FOI) 0.1957 | | |

| **Table S5. Predictors of diverse *Bartonella* spp. infections in ectoparasite arthropods, from GLM** | | | | | |
| --- | --- | --- | --- | --- | --- |
| ***Bartonella* spp. in arthropod ^†^** | **Predictor** | **Slope** | **Lower CI^††^** | **Upper CI^††^** | **P-value** |
| *B. elizabethae 1***^†††^** | Rat Seq: *B. elizabethae 1* | 21.42 | -368.22 | 411.05 | 0.914 |
|  | Rat Seq: *B. elizabethae 2* | -1461 | -93009600 | 93006680 | 0.999 |
|  | Rat Seq: *B. phoceensis 1* | 22.19 | -370.60 | 414.98 | 0.912 |
|  | Rat Seq: Negative | 1.69 | -391.87 | 395.24 | 0.993 |
|  | Ecto Type: *S. fonquerniei* | 21.82 | -356.31 | 399.95 | 0.910 |
|  | Ecto Type: *X. cheopsis* | 21.63 | -379.76 | 384.08 | 0.991 |
|  | Ecto Type: *E. gallinacea* | -340.6 | -37970750 | 37970070 | 1.000 |
|  | Ecto Type: *Polyplax* spp. | -999.8 | -93009140 | 93007140 | 1.000 |
|  | Ecto Type: Mesostigmatid | 0.4033 | -383.71 | 384.52 | 0.998 |
| *B. elizabethae 2***^†††^** | Rat Seq: *B. elizabethae 1* | -2.42 | -126.70 | 121.86 | 0.970 |
|  | Rat Seq: *B. elizabethae 2* | 71.03 | -93008070 | 93008210 | 1 |
|  | Rat Seq: *B. phoceensis 1* | -2.46 | -126.55 | 121.63 | 0.969 |
|  | Rat Seq: *B. rattimassiliensis 1* | -564.71 | -51029030 | 51027900 | 1 |
|  | Ecto Type: *S. fonquerniei* | 31.36 | -486192 | 486255 | 1 |
|  | Ecto Type: *X. cheopsis* | 29.66 | -486194 | 486253 | 1 |
|  | Ecto Type: *E. gallinacea* | -4999.76 | --38919260 | 38909260 | 1 |
|  | Ecto Type: *Polyplax* spp. | 11.60 | -18739220 | 18739240 | 1 |
|  | Ecto Type: Mesostigmatid | 11.09 | -6162418 | 6162440 | 1 |
| **^†^**Since only one arthropod ectoparasite (louse) each sequenced positive for *B. phoceensis 1* and *B. rattimassiliensis 1,* respectively, we did not include these genotypes in this GLM analysis. **^††^**Confidence intervals were computed for the slope of each interaction as slope ±1.96*standard error. **^†††^**Challenges from zero counts in our dataset forced us to change the reference level in our rat sequence predictor between the two analyses (rat seq reference was *B. rattimassiliensis 1* for the *B. elizabethae 1* GLM and “negative” for the *B. elizabethae 2* GLM). | | | | | |

| **Table S6. *Bartonella* species-specific prevalence in blood-feeding arthropods** | | | | | |
| --- | --- | --- | --- | --- | --- |
|  |  | ***Bartonella* spp. prevalence by species [# pos (%)]** | | | |
| **Ectoparasite** | **# Seq^†^** | *B. elizabethae 1* | *B. elizabethae 2* | *B. phoceensis 1* | *B. rattimassiliensis 1* |
| *S. fonquerniei* | 24**^†††^** | 15 (62.5) | 5 (20.8) | 0 (0) | 0 (0) |
| *X. cheopsis* | 8 | 1 (12.5) | 1 (12.5) | 0 (0) | 0 (0) |
| *E. gallinacea* | 12**^††^** | 0 (0) | 0 (0) | 0 (0) | 0 (0) |
| *Polyplax spp.* | 2 | 0 (0) | 0 (0) | 1 (50) | 1 (50) |
| *Haemaphysalis* spp. | 6 | 0 (0) | 0 (0) | 0 (0) | 0 (0) |
| Mesostigmatid | 6**^††^** | 1 (16.7) | 0 (0) | 0 (0) | 0 (0) |
| **^†^**All ectoparasites highlighted here were recovered from a *different* rat host. **^††^**In two instances of *E. gallinacea* infestation and one of Mesostigmatid mite infestation, we bulk assayed 10, 15, and 15 ectoparasites (respectively) of the same type from the same host after the first arthropod tested negative. All subsequently assayed ectoparasites were negative, as well, in contrast to the one instance**^†††^** in which we assayed five *S. fonquerniei* from a *B. elizabethae 1-*positive rat host; in this case, all five sampled fleas were positive with *B. elizabethae 1.* | | | | | |

**Supplementary Figures**


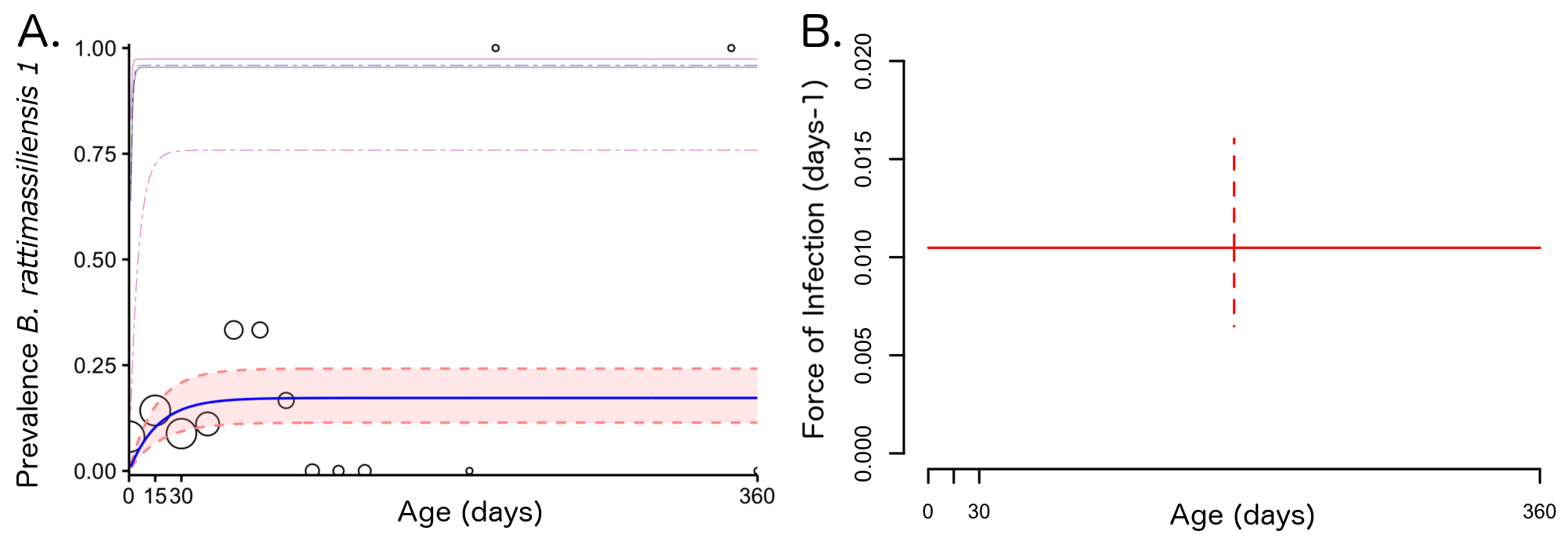


**Figure S1.** Age-prevalence (A) and force of infection (B) for sampled *Rattus rattus* infected with *B. rattimassiliensis 1* in the alternative, one age class SIS model form (model fits between this model and the 3-age-class SI version exhibited in Fig 2G/H of the main text were indistinguishable via LRT; see Table S1). As in Fig 2 in the main text, open circles in (A) signify age-stratified prevalence from the data binned over 15-day intervals, and circle size correlates to sample size within each bin. The blue line represents the expected cumulative proportion infected by age from the best fit model. Pink shading encompasses the 95% confidence interval as determined via partial profile likelihood, and faint background lines depict predicted prevalence from the more relaxed version of the model allowing for deviations in *λ(a)* by sampling site (navy solid = Ankazobe Outside, navy dashed = Ankazobe Inside; fuchsia solid = Ranomafana Outside; fuchsia solid = Ranomfana Inside). Confidence intervals for site-specific FOI estimates are listed in Table S2.


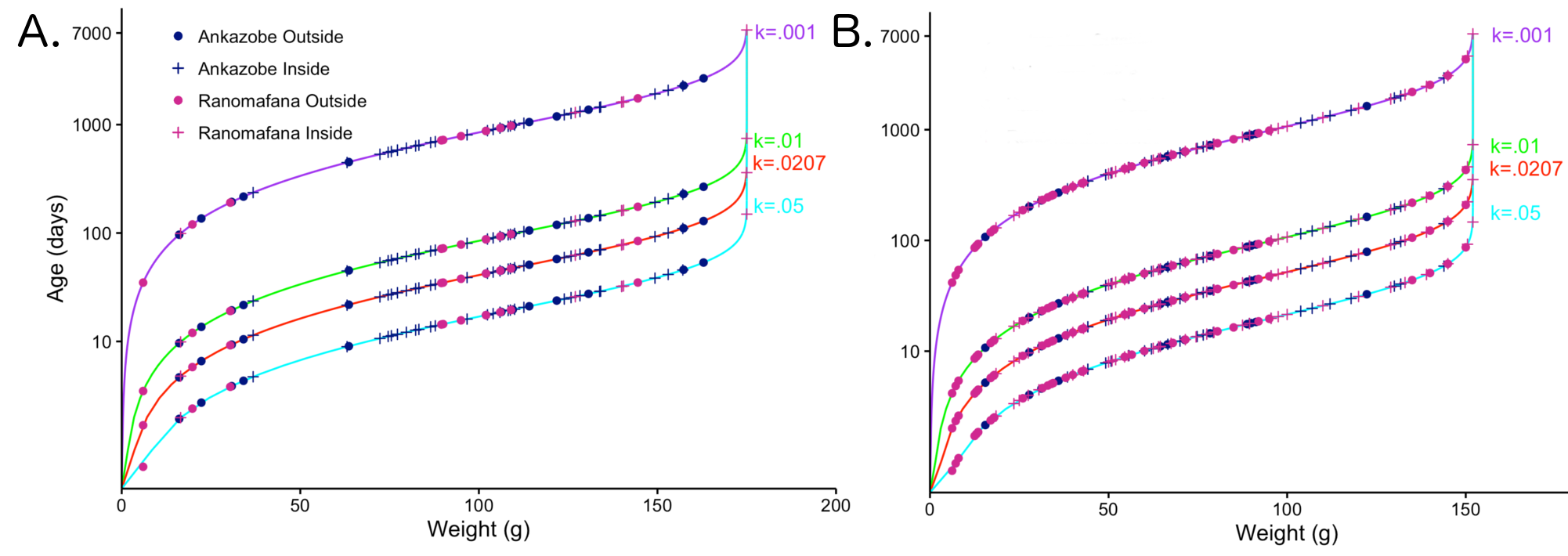


**Figure S2.** Age-for-weight relationships for captured *R. rattus* (A) males and (B) females as estimated from the von Bertalanffy equation (Ricker, 1979): *W(t) = W(1-e^-k(t-t0)^)* in which *W* represents the highest weight class rodent in the data subset. Mirroring Figure 1 in the main text, for males, this was a rodent of 175 g and for females, this was a rodent of 152 g. Data from Ankazobe sites are depicted in navy (outside sampling sites= circles, inside sampling sites = pluses) and from Ranomafana in fuchsia. The labeled curves in each figure depict the age estimations for a range of values of *k,* a parameter describing the maturation rate of the modeled species. The color of each curve matches the color of the *k*-value used to produce it (purple: *k*=.001, green: *k*=.01, red: *k*=.0207, cyan: *k*=.05). Age in days (y-axis) is plotted on a log-10 scale.
