# Supplementary material for: Elucidating transmission dynamics and host-parasite-vector relationships for rodent-borne Bartonella spp. in Madagascar
Source: Epidemics. 2017 Sep;20:56–66. doi: 10.1016/j.epidem.2017.03.004 (PMC5608689; doi:10.1016/j.epidem.2017.03.004)
Supplement: Supplementary file 2 [file mmc2.docx]

**S1 Dataset Legend**

Columns for the supplementary data file are coded as follows:

**Location.Subsite**

- Location:
  - 1=Andreba
  - 2=Ambaninmontana
  - 3=Maharidaza
  - 4=CVB
  - 5=Ranomafana Village
  - 6= Ranomafana Forest
- Sub-Site:
  - 1=rice_paddy
  - 2=village
  - 3=CVB
  - 4=forest

**Sex**

- 0=Male
- 1=F
- 2=unidentified

**Rat nuoG PCR**

- 0= Negative
- 1= *B. elizabethae 1*
- 2= *B. elizabethae 2*
- 3= *B. phoceensis 1*
- 4= *B. rattimassiliensis 1*
- 5 = *B. tribororum 1*
- 6 = Novel *Bartonella* sp.
- 7 = Not Tested

**Ectoparasite Type for PCR**

- 0 = None Tested
- 1 = Synopsyllus
- 2= Xenopsylla
- 3 = Echidnophaga
- 4=Polyplax
- 5 = Haemaphysalis ticks
- 6=Mesostig

**Ectoparasite nuoG PCR**

- 0= Negative
- 1= *B. elizabethae 1*
- 2= *B. elizabethae 2*
- 3= *B. phoceensis 1*
- 4= *B. rattimassiliensis 1*
- 5 = *B. tribororum 1*
- 6 = Novel *Bartonella* sp.
- 7 = Not Tested
